# The Mouse Genome Database (MGD): facilitating mouse as a model for human biology and disease

**DOI:** 10.1093/nar/gku967

**Published:** 2014-10-27

**Authors:** Janan T. Eppig, Judith A. Blake, Carol J. Bult, James A. Kadin, Joel E. Richardson, The Mouse Genome Database Group

**Affiliations:** The Jackson Laboratory, 600 Main Street, Bar Harbor, ME 04609, USA

## Abstract

The Mouse Genome Database (MGD, http://www.informatics.jax.org) serves the international biomedical research community as the central resource for integrated genomic, genetic and biological data on the laboratory mouse. To facilitate use of mouse as a model in translational studies, MGD maintains a core of high-quality curated data and integrates experimentally and computationally generated data sets. MGD maintains a unified catalog of genes and genome features, including functional RNAs, QTL and phenotypic loci. MGD curates and provides functional and phenotype annotations for mouse genes using the Gene Ontology and Mammalian Phenotype Ontology. MGD integrates phenotype data and associates mouse genotypes to human diseases, providing critical mouse–human relationships and access to repositories holding mouse models. MGD is the authoritative source of nomenclature for genes, genome features, alleles and strains following guidelines of the International Committee on Standardized Genetic Nomenclature for Mice. A new addition to MGD, the Human–Mouse: Disease Connection, allows users to explore gene–phenotype–disease relationships between human and mouse. MGD has also updated search paradigms for phenotypic allele attributes, incorporated incidental mutation data, added a module for display and exploration of genes and microRNA interactions and adopted the JBrowse genome browser. MGD resources are freely available to the scientific community.

## INTRODUCTION

The Mouse Genome Database (MGD, http://www.informatics.jax.org) (1–3) serves the international research community as the central resource for integrated genomic, genetic and biological data on the laboratory mouse. Since its inception 25 years ago, MGD has served as the authoritative source for mouse genes, genome features, mutations and strain nomenclature. In recent years, it has become the source for the unified catalog of mouse genome features, the comprehensive set of Gene Ontology (GO) annotations (functional associations) for mouse protein-coding genes, the comprehensive source for mouse phenotype annotation using the Mammalian Phenotype (MP) Ontology and the connection between mouse genotypes and the human diseases that they model.

The catalog of genome features includes genes, functional RNAs, quantitative trait loci and a changing group of heritable loci defined by phenotypic observations that form the basis upon which mouse biology is connected to the genome. MGD develops and maintains the gene catalog by integrating computational and manually curated genome annotations from NCBI, Ensembl and Havana into a single, non-redundant resource. MGD is also a member of the Consensus CDS project to provide a curated list of high-confidence protein-coding genes for which annotations from different providers are consistent (4). MGD curators work in collaboration with these resource groups to continually improve the mouse genome assembly and genome feature assignments.

Functional annotations for mouse protein-coding genes are curated and maintained using the GO. MGD, in addition, contributes to the development of GO through review and addition of terms to the Ontology, as well as contributions to its overall structure (5). MGD is the authoritative source for mouse GO annotations, providing its data to the combined GO Consortium site. Mouse GO annotations, based on the extensive experimental data available for mouse through MGD, contribute to phylogenetically based inferred annotations in a wide variety of species with little functional experimental data.

MGD is increasing its representation of phenotype and disease models to improve support for translational research. MGD captures the comprehensive set of mouse mutations (spontaneous, induced and genetically engineered) and available data about these mutations’ phenotypic effects. Data are curated and integrated from the biomedical literature, researcher submissions and large-scale projects. Phenotypic data are standardized using the MP Ontology (6) and genotypes that model human diseases are associated with terms from the Online Mendelian Inheritance in Man (OMIM) (7) where those genotypes recapitulate the human conditions.

MGD is the primary component and underpinning data core for a collection of mouse genome data resources that constitute the Mouse Genome Informatics (MGI) resource. Additional components of MGI include the Gene Expression Database for Mouse Development (8), the Mouse Tumor Biology Database (9), the CrePortal (10), the MouseCyc database of biochemical pathways (11) and the International Mouse Strain Resource (IMSR) (12).

Table 1 summarizes the current data content for MGD.

## NEW FEATURES AND IMPROVEMENTS

In the last year, MGD has added new features, improved others and integrated new data sets. These include: the development of the Human–Mouse: Disease Connection interface, which enables the exploration of relationships between human and mouse genes and phenotypes, and their connections to known human diseases; the addition of explicit gene–gene and gene–allele relationships that allow users access to interaction data, and that reveal genomic mutations encompassing multiple genes; the addition of incidental mutations data sets discovered through genome sequencing of N-ethyl-N-nitrosourea (ENU) mutagenized mice and their progeny; key improvements in phenotype querying that speed search performance and provide more precise searching for alleles; and implementation of the JBrowse (13) genome browser.

### Human–Mouse: Disease Connection

**Figure 1.**
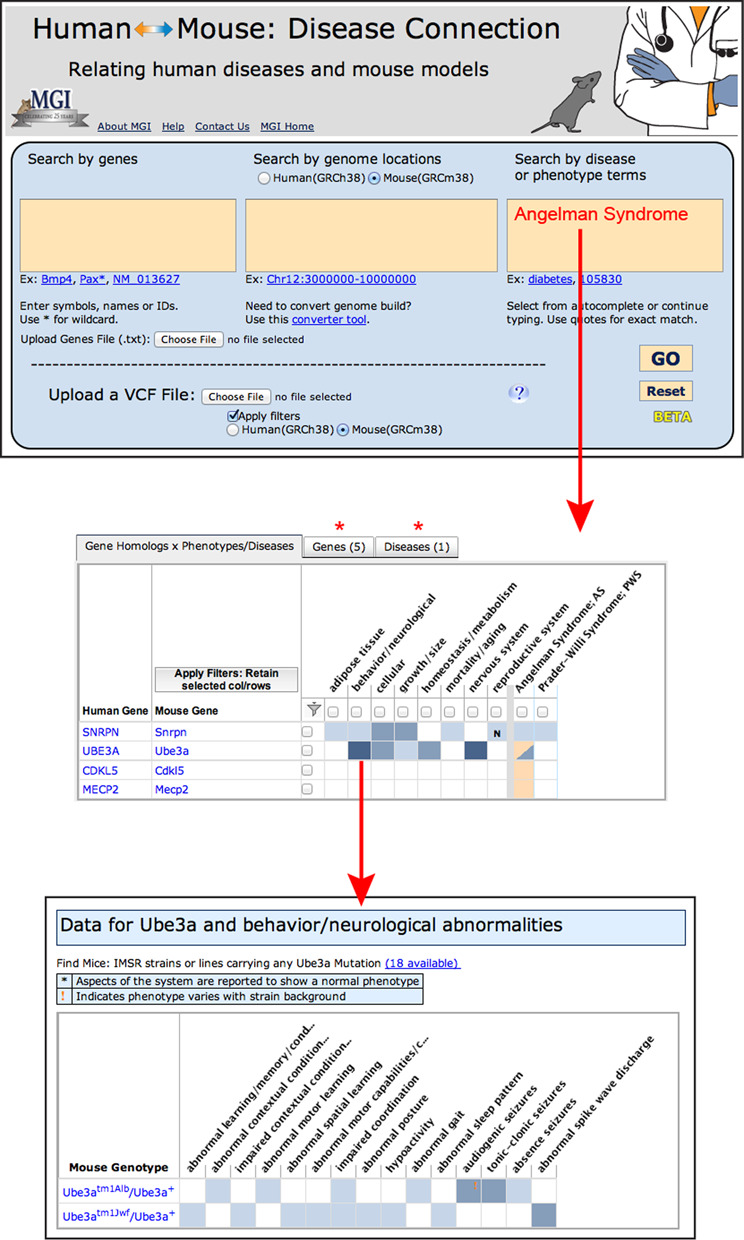
Human–Mouse: Disease Connection (HMDC). The top panel shows the upper portion of the HMDC homepage. Searches can be initiated using human or mouse gene(s), location(s) or disease/phenotype terms. Alternatively VCF files or files of gene symbols or IDs can be submitted as search parameters. The disease/phenotype search box has an autocomplete feature allowing the user to choose the exact term desired. In this example Angelman Syndrome was selected. The results (middle panel) are presented in grid format, listing genes associated with Angelman Syndrome in human or mouse in the left-most columns. Grid colors representing mouse data are blue and human data are orange, with color intensity being darker for more annotations. Phenotype and disease terms are indicated in the columns on the right-hand side of the grid. Note that both human and mouse homologs (*UBE3A* and *Ube3a*, respectively) are associated with the disease. In addition, human *CDKL5* and *MECP2* are associated human genes and *Snrpn* is an associated mouse gene. This might suggest additional mouse models could be created by mutating mouse genes *Cdkl5* or *Mecp2*; and that other potential human mutations in *SNRPN* might be examined as an Angelman Syndrome candidate gene. For genes that have known models in mice (*Snrpn* and *Ube3a*) a phenotype profile is provided. The red asterisk (*) indicate tabs that display data in a tabular format by genes or by diseases. Each colored cell within the grid is interactive and clicking on a cell leads to further details (bottom panel).

The Human–Mouse: Disease Connection (http://www.diseasemodel.org) is a translational tool providing simultaneous access to human–mouse genomic, phenotypic and genetic disease information (Figure 1). Researchers can explore phenotypes and disease relationships, identify candidate genes and evaluate mouse mutants displaying a spectrum of indicative phenotypes. Within the web display, links to supporting mouse model publications and to repositories providing mouse resources make this tool informative as well as exploratory.

Three primary approaches give users flexibility to search from a human or mouse perspective, using (i) genes or gene IDs for either species, (ii) genome coordinate(s) for either species and (iii) mouse phenotype or human disease terms. Data can also be uploaded from Variant Call Format (VCF) files or text files of gene IDs or gene symbols. Thus, exploration can begin with a single gene or set of genes, a region for Quantitative Trait Loci (QTL), multiple deletion regions or using phenotype/disease searches, such as ‘Crouzon Syndrome’, ‘neurofibromatosis’, ‘exencephaly’ or ‘cardiomyopathy’.

All search methods initially return an interactive grid that shows a visual overview of results and facilitates comparison of phenotypes and diseases across multiple genes, phenotypes and diseases. The grid features color cues reflecting depth of annotated human and mouse data, and grid cells are active links leading to more detailed information, including availability of mouse models from repositories worldwide. Alternate web displays with gene and disease-focused information are a single click away.

Integration of mouse and human data related to gene homologs, genomic locations, mutations, phenotypes and diseases is ongoing in MGD. Currently (1 September 2014) MGD includes over 1320 OMIM-defined human genetic diseases with at least one experimentally defined mouse model, with over 4450 total mouse models; and data on mouse phenotypes in more 52 570 unique genotypes. These data combine existing MGD data for mouse genome coordinates, gene identity, phenotypes and mouse models originating via curation of biomedical literature, investigator data submissions and downloads from major data providers. The associations of human genes with human diseases are obtained from NCBI (combining data of OMIM, GeneReviews, GeneTests and NCBI-curated information). Newly available phenotypic data from the systematic screens of knockout mutations analyzed in the International Mouse Phenotype Consortium (14) project will further augment phenotype–genotype associations for mouse mutations.

### JBrowse genome browser

**Figure 2.**
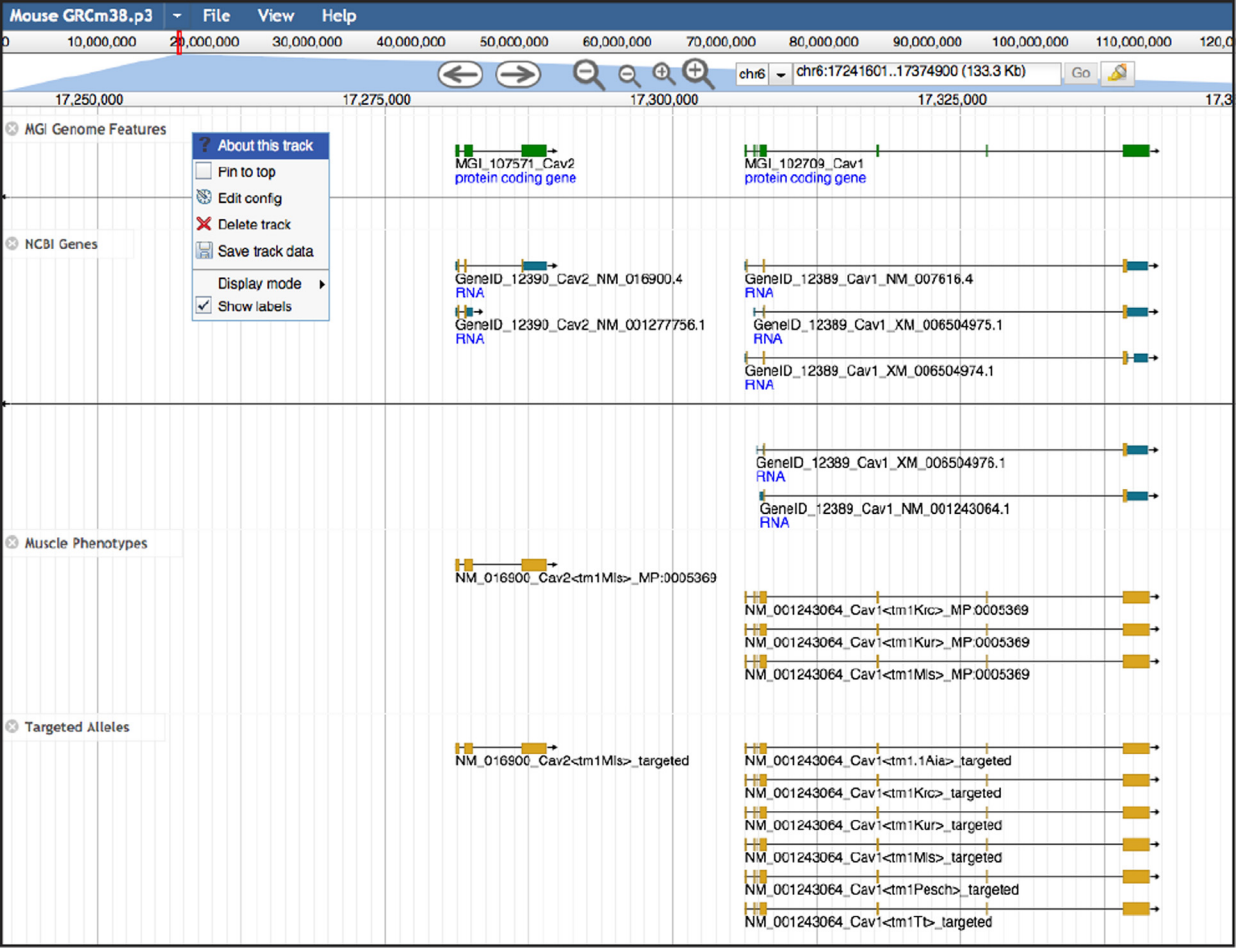
Mouse genome annotation browser implemented in JBrowse. Example of the allele and phenotype annotations for mouse *Cav2* and *Cav1* genes. The MGI track displays unified mouse gene catalog contents. Tracks from external genome annotation groups such as NCBI provide details regarding transcriptional isoforms of mouse genes. Track controls allow users to customize aspects of the display and to download data from the JBrowse tracks.

A primary mission of the MGD project is to integrate biological annotations associated with mouse genes and proteins with large-scale sequence data sets and with the reference genome for the laboratory mouse. One of the best mechanisms for exploring these data is through interactive graphical displays. We have deployed a powerful new browser called JBrowse (13) (Figure 2). JBrowse features significantly improved speed for rendering annotation tracks in a web browser window compared to previous genome browser software. The architecture of JBrowse allows the MGD team to update data more frequently and to make available to the community larger data files that are typical of most genome-centric experiments. Even very dense data sets such as single nucleotide polymorphism (SNPs) can be rendered without significant delay. Users can easily download the annotations in a particular genome region. Callback functionality has been expanded to give software developers the ability to add custom context menus and visualizations on a per track basis. The MGD installation of JBrowse includes data we acquire from external annotation providers as well as MGD-specific annotations for phenotype and function. Since multiple genomes can be contained within a single JBrowse instance we have included the human genome reference assembly and NCBI's human genome annotations.

### Genome feature–genome feature relationships

MGD has developed new infrastructure to support genome feature relationships. In its initial implementation, two specific relationship types are represented. First, genome feature cluster relationships link genes that belong to a cluster located in a genomic region and second, genome feature–genome feature interactions that are designed to show how products of one gene are influenced by others. The initial interaction data sets incorporated include predicted and verified gene targets of microRNAs.

#### Genome feature cluster relationships

**Figure 3.**
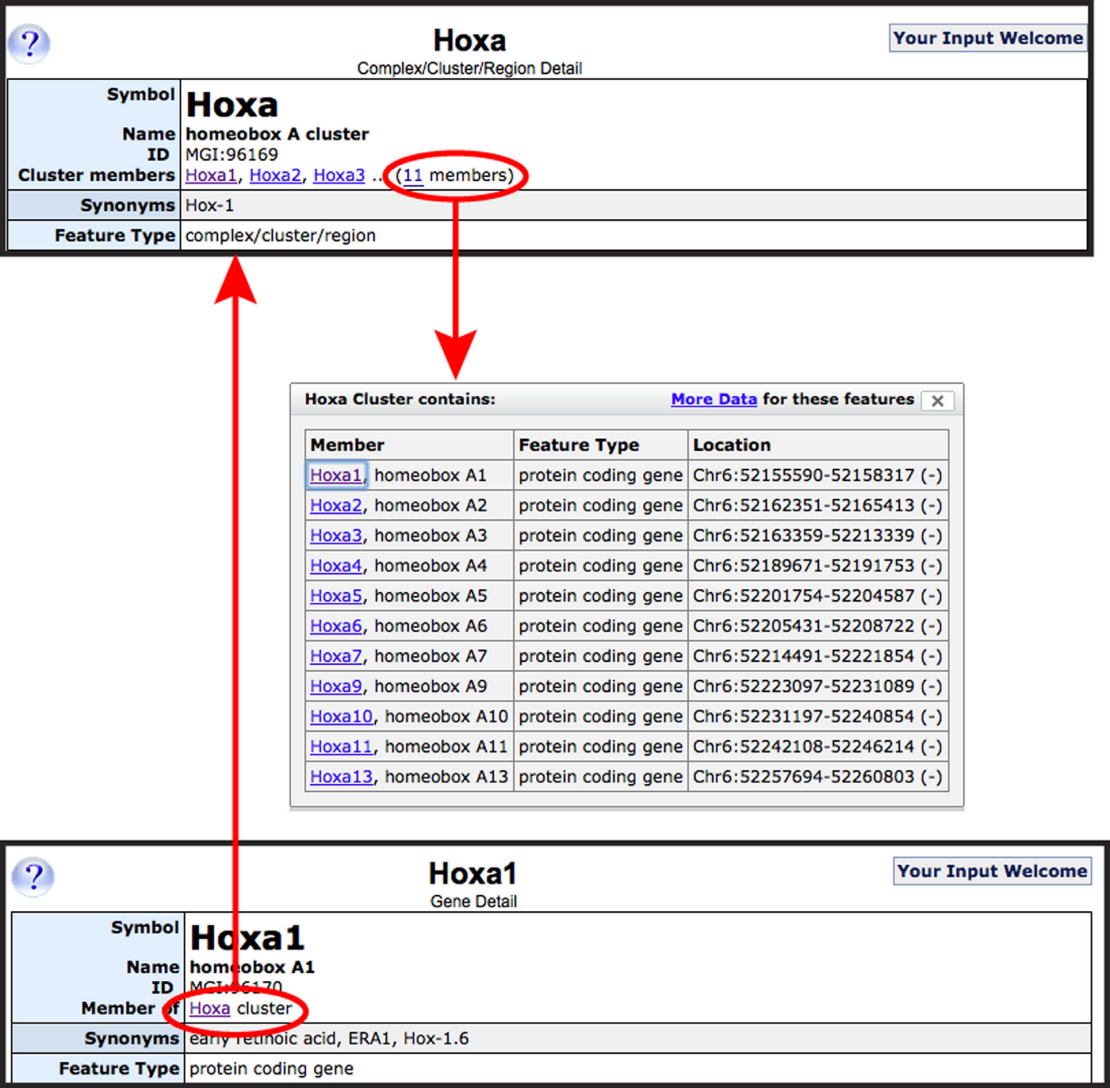
Genome feature–genome feature cluster relationship. Top panel shows the upper portion of the homeobox A cluster detail page. Cluster members are now indicated, with a link to the full list of genes in the cluster membership. Each cluster member gene page (bottom panel) shows what cluster that gene is a member of, with a link to the cluster's detail page.

Closely related genome features are sometimes found in clusters in the genome. Likewise genomic changes may affect one or multiple members of a cluster. One example is the *Hoxa* cluster, comprising 11 individual *Hoxa#* genes spanning a 105 kb region of Chromosome 6. Specific genetically engineered mutations have been designed to remove one or several of the individual cluster members. The cluster genome feature detail page now links to a full list of cluster members; and the gene detail page for each cluster member displays its cluster membership (Figure 3).

#### Genome feature–genome feature interactions

**Figure 4.**
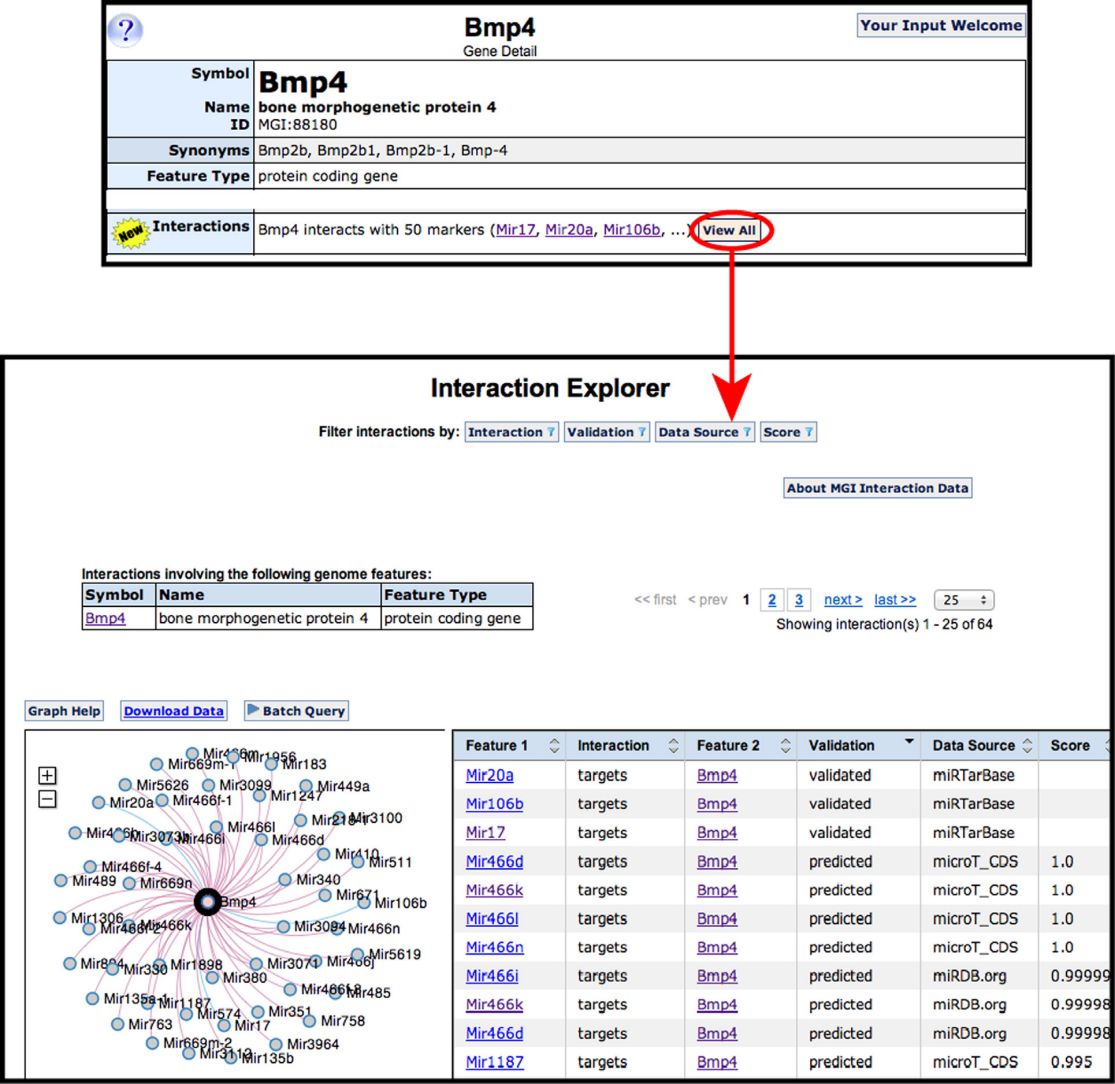
Interaction Explorer. On the *Bmp4* gene detail page (top panel), a new ‘Interactions’ ribbon has been added. The initial sets of interaction data incorporated by MGD are interactions of genes with microRNAs. The *Bmp4* gene interacts with 50 genome features that can be viewed by selecting the ‘View All’ button. The Interaction Explorer page (bottom panel) graphically and dynamically displays interactions on the left and provides a tabular view on the right. By using the filtering and sorting options, users can limit the number of interactions shown. The graphical view changes in response to such filtering and can be made larger or smaller to aid viewing. The selected marker (here, *Bmp4*) is shown in the center of the graph display with blue lines connecting validated interactions and red lines connecting predicted interactions.

The first relationship to be supported for genome feature–genome feature interactions in MGD describes predicted and verified gene targets of known mouse microRNAs. Data include both validated and predicted interactions from miRTarBase (15), microt-cds (16) and Pictar (17). When viewing a gene feature detail page or a microRNA detail page, a new ribbon is seen, labeled ‘Interactions’. For example, the gene page for the *Bmp4* gene indicates that it interacts with 50 microRNAs. One of the validated microRNA interacting with *Bmp4* is *Mir106b*, which interacts with 4597 other mouse genome features. Choosing to ‘View All’ from these respective pages takes the user to the new Interaction Explorer page (Figure 4) that presents the gene–microRNA interactions as an interactive graphical display and a table of validated and predicted interactions. The table can be sorted or filtered in various ways by the user, and these actions are dynamically reflected in the graphical Explorer. Results can also be downloaded as a file.

**Table 1.** Summary of MGD data content (1 September 2014)

Type of data	Counts for data
Number of genes with protein sequence data	24 613
Number of mouse genes with human orthologs	17 055
Number of mouse genes with rat orthologs	18 461
Number of protein-coding genes with functional (GO)^a^ annotations	24 320
Total number of GO^a^ annotations	291 605
Total number of mutant alleles (in mice or ES cells)	751 981
Number of mutant alleles (in mice)	40 276
Number of genes with one or more mutant alleles	21 859
Number of genes with one or more phenotypic alleles^b^ (in mice)	12 619
Number of genes with targeted alleles	16 645
Number of genes with targeted alleles (in mice)	9225
Number of genotypes with phenotype annotation (MP)^c^	52 570
Total number of MP^b^ annotations	273 105
Human diseases with one or more mouse models	1323
Number of QTL	4859
Number of references in the MGD bibliography	206 753

^a^GO = Gene Ontology.

^b^Phenotypic alleles are those that result in some phenotypic abnormality when present in heterozygous or homozygous state.

^c^MP = Mammalian Phenotype Ontology.

### Gene–allele relationships

The vast majority of mutations in mouse, whether spontaneous, induced or genetically engineered, are changes to the sequence of a unique gene. However, some mutational changes are genomic in nature, for example, involving contiguous multi-gene deletions in the simplest case, to very complex rearrangements, involving duplications, deletions or fusions of genomic components.

**Figure 5.**
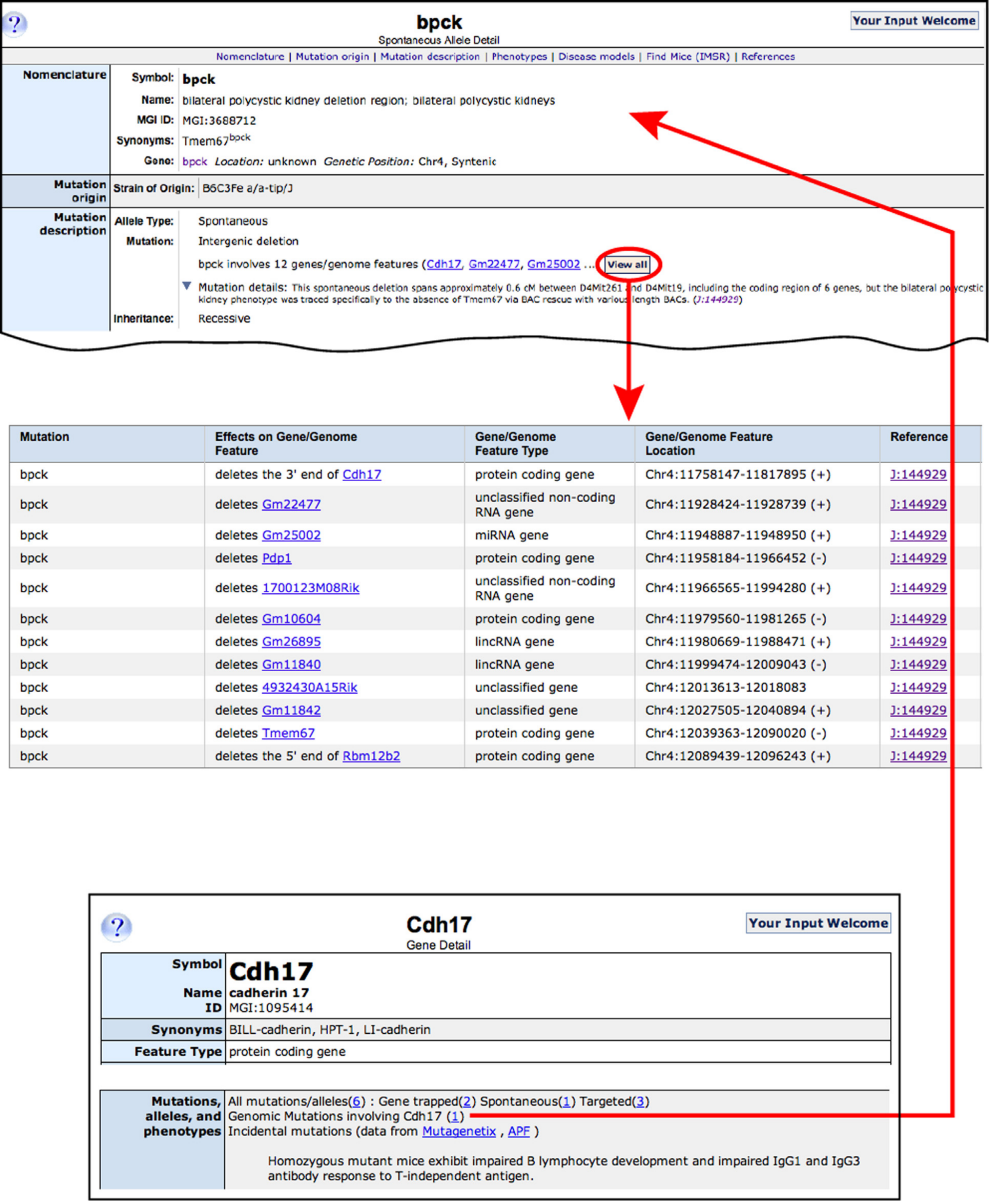
Gene–allele relationships. MGD now represents the components of complex mutations. For a given complex mutation (here, *bpck*), the individual genes or genome features mutated are viewable, along with the type of change that each has undergone (top and middle panels). Conversely, any gene detail page for a single genome feature component of a complex mutation (here, *Cdh17* is a component of the complex mutation *bpck*) indicates the existence of its participation in ‘genomic mutations’ that include that particular gene.

MGD has revised its schema to represent complex mutations. Each ‘genomic mutation’ is captured as a list of contributing genomic components and the changes to each component relative to its native state. Figure 5 shows the components of one such complex mutation, *bpck*, bilateral polycystic kidney deletion region, which affects a genomic region spanning 12 genes/genome features. Previously, the *bpck* mutation was represented in a unique mutation record, but was not associated with the 12 genes/genome features affected by the mutation. Now *bpck* is associated with each of the component genes it includes, and each component gene is associated with the ‘genomic mutation’ *bpck*.

### Incidental mutations

Incidental mutations are those discovered in the course of identifying a causative mutation associated with a phenotype of interest. Usually, these mutations are uncovered during the analysis of sequence data obtained for animals from ENU mutagenesis experiments. Additional new mutations are often discovered that are unrelated to the primary phenotype (ENU is known to be a point-mutagen that can cause many mutations in the experimentally treated males (18)). These incidental mutations are similar in concept to the human incidental mutations discovered during sequencing for the disease causing mutation. Incidental mutations are sequence-identified, but with unknown effects and unrelated to the primary cause of phenotype or disease at hand.

MGD now provides access to incidental mutations from sequencing of animals associated with ENU studies. These incidental mutations provide an additional source of mutational variation that is useful for (i) genes in which no or few mutations are known and (ii) genes in which one would like to have an allelic series of mutations to study. Conversely, researchers interested in the primary ENU mutation that has been identified and studied need to be aware of additional mutations potentially segregating in the background of the ENU mutant-bearing strain that could produce confounding results in phenotypic analyses.

**Figure 6.**
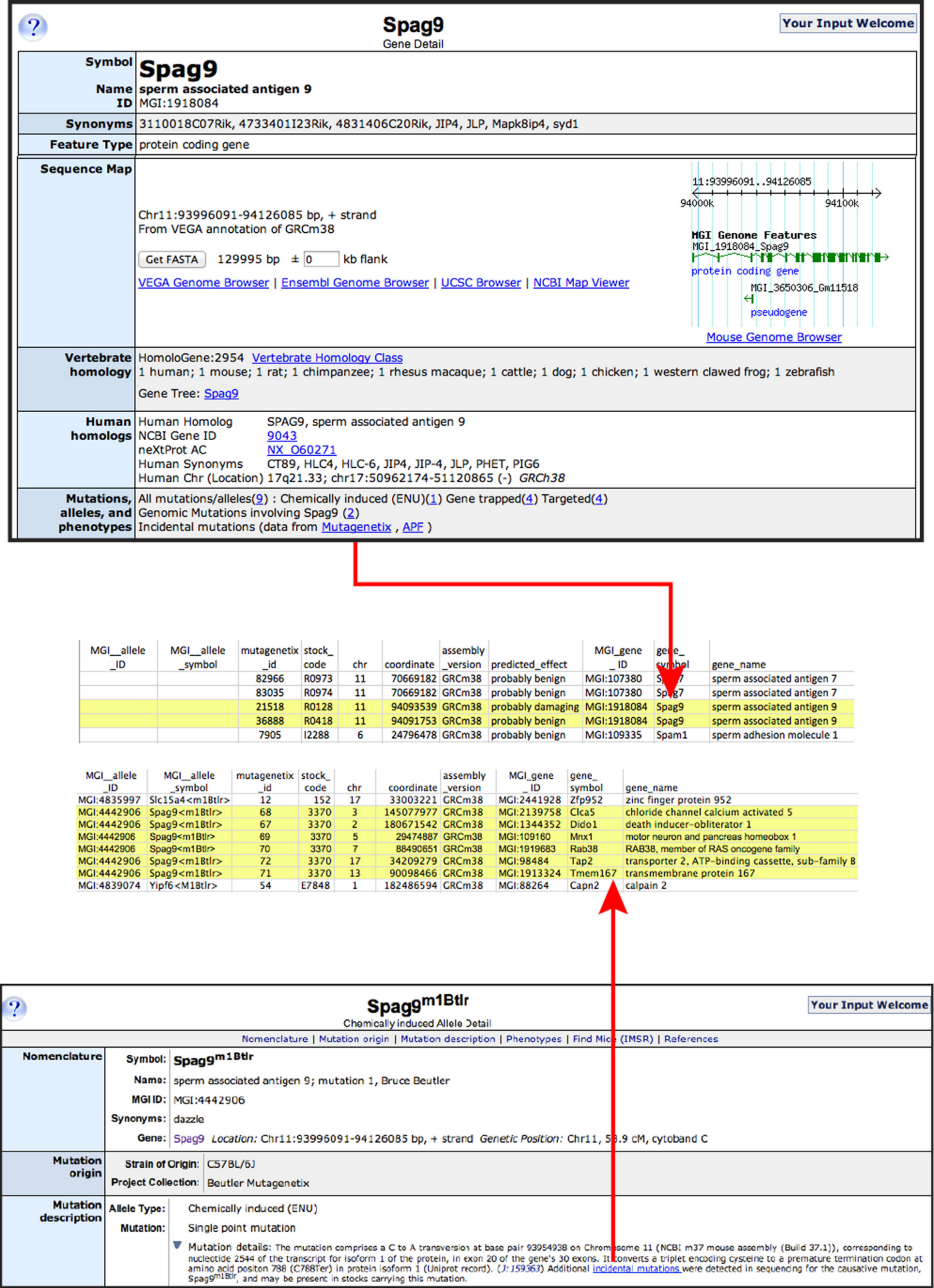
Incidental Mutations from ENU experiments. Incidental mutations are accessed from the Gene Detail Page or Allele Detail Page. The top panel shows the *Spag9* gene page with a link to two incidental mutations in this gene from the Mutagenetix mutation collection. *Spag9* mutations were discovered in two mutant stocks from this collection. The bottom panel shows the *Spag9^m1Btlr^* allele detail page. Six incidental mutations were detected in the stock carrying this mutation.

To date, MGD provides incidental mutation data from the Mutagenetix project (19) and the Australian Phenomics Facility (20). We are currently working to import data from the Cardiovascular Development Consortium mutagenesis program (21), and additional sets of incidental mutation data will follow. Links to these data are found on MGD's Gene Detail Pages in the Mutations, Alleles and Phenotype ribbon and for incidental mutations occurring in a particular ENU mutation stock, links are provided on the Mutant Allele Detail Pages in the Mutation Details section (Figure 6).

### Allele attributes and project collections

**Figure 7.**
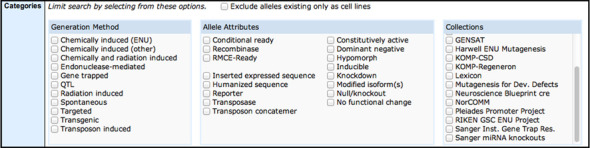
Allele attributes and project collections. Screen shot of the Categories ribbon from the Phenotypes, Alleles & Disease Models Query Form (http://www.informatics.jax.org/allele/). When searching for alleles with specific characteristics a generation method may be selected, one or more allele attributes may be selected, and membership of an allele in a project collection may be selected. For example, one might select the generation method ‘Chemically induced (ENU)’ and attribute ‘Hypomorph’ to find those ENU mutations that show partial function. A selection from the project collections attribute would further restrict one's query to those alleles generated in the project(s) selected.

The MGD schema and web pages reflecting data on allele categories, attributes and project collections have been re-implemented and expanded. Formerly allele categories included multiple concepts within a single term. For example, the categorical term targeted (knock-out) referred to mutant alleles that were generated by gene targeting techniques and were functionally null. This term now is represented as a *Generation Method* of targeted and an *Allele Attribute* of null/knockout. The change separates the generation method and attributes concepts and allows users to consider multiple attributes simultaneously (Figure 6), for example reporter and null/knockout—if the targeted mutation includes a LacZ reporter, as well as ablating gene function. The additional category of project collections is an enhancement to assist in accessing all mutant alleles from a large-scale project in a single search, for example all of the mutant alleles created by the Pleiades Promoter Project (22) or by the B2B/CvDC (Bench-to-Bassinet/Cardiovascular Development Consortium) Project (21) (Figure 7).

## OTHER INFORMATION

### Mouse gene, genome feature, allele and strain nomenclature

MGD is the authoritative source for the international scientific community for nomenclature for mouse genes, genome features, alleles, mutations and strains. Guidelines set by the International Committee on Standardized Genetic Nomenclature for Mice (http://www.informatics.jax.org/nomen) are implemented through MGD. Official nomenclature and IDs are distributed worldwide through the MGD web site and through regular data exchanges with other bioinformatics resources. MGD actively promotes adherence to nomenclature standards in publications and online sites and works with journal editors and consortia to ensure use of nomenclature standards. In addition, MGD, the Human Gene Nomenclature Committee (23) and the rat genome database (24) collaborate to co-assign genome feature symbols that are consistent for orthologs across species. To contact the MGD nomenclature coordinator for assistance with nomenclature, use email: nomen@jax.org.

### Bulk and programmatic data access

MGD provides access to its data by a number of commonly used tools, including Web Services, BioMart and MouseMine (see links on http://www.informatics.jax.org/software.shtml). In addition, bulk data sets are available as FTP reports (ftp://ftp.informatics.jax.org/pub/reports/index.html) and via the MGD Batch Query tool (http://www.informatics.jax.org/batch), which allows users to customize data sets.

### Electronic data submission

MGD accepts contributed data sets from individuals and organizations for any type of data maintained by the database. The most frequent types of contributed data are mutant and phenotypic allele information originating with the large mouse mutagenesis centers and strain data from repositories that contribute to the IMSR (http://www.findmice.org) (12). Each electronic submission receives a permanent database accession ID. All data sets are associated with their source, either a publication or an electronic submission reference. Details about data submission procedures can be found at http://www.informatics.jax.org/submit.shtml.

MGD also provides a ‘Your Input Welcome’ link in the upper right hand corner of gene and allele detail pages. Users are encouraged to submit corrections and additions to data through this page. MGD staff will follow up if there are questions about the submission.

### Community outreach and user support

The MGD resource has full time staff members who are dedicated to user support and training. Members of the User Support team can be contacted via email, web requests, phone or Fax.

- World wide web: http://www.informatics.jax.org/mgihome/support/mgi_inbox.shtml.
- Facebook: https://www.facebook.com/mgi.informatics.
- Twitter: https://twitter.com/mgi_mouse.
- Email access: mgi-help@jax.org.
- Telephone access: +1 207 288 6445.
- Fax access: +1 207 288 6830.

MGD User Support staff are available for on-site help and training on the use of MGD and other MGI data resources. MGD provides off-site workshop/tutorial programs (roadshows) that include lectures, demos and hands-on tutorials and can be customized to the research interests of the audience. To inquire about sponsoring a MGD roadshow, email mgi-help@jax.org.

On-line training materials for MGD and other MGI data resources are available as FAQs and on-demand help documents.

### Other outreach

MGI-LIST (http://www.informatics.jax.org/mgihome/lists/lists.shtml) is a moderated and active email bulletin board for the scientific community supported by the MGD User Support group. The MGI-LIST has over 1800 subscribers. A second list service, MGI-TECHNICAL-LIST, is maintained for technical information for software developers and bioinformaticians accessing MGI data, using APIs and making links to MGI.

## IMPLEMENTATION AND PUBLIC ACCESS

The master internal MGD database resides in a normalized relational database and is the workplace for integration of MGD data. This database is optimized for data loading, curation and integration processes. As data are prepared for the weekly public release, they are migrated to a public database instance in PostgreSQL that is denormalized and supplemented by an MGD set of Solr/Lucene (http://lucene.apace.org/solr) indexes. This public instance of MGD has excellent performance qualities for supporting searches and web displays. Keeping distinct versions of MGD for internal data loading, curation and integration and for public access on the web via a denormalized search-optimized version also helps us to manage the impact of changes required to either the internal or public MGD versions.

MGD provides free public access to data from http://www.informatics.jax.org. The web interface provides a simple ‘Quick Search’, available from all web pages in the system and is the most used first entry point for users. Various query forms are provided that allow more precise parameter searching. For example, using ‘prostaglandin’ as the keyword in the ‘Quick Search’ box returns 232 genome features (as of September 2014). In contrast, using the Genes and Markers Query form and entering ‘prostaglandin’ in the gene name box, choosing feature type of protein-coding gene and location of Chr 14 returns two results. Query forms for specific parameter searching are available for Genes and Markers; Phenotypes, Alleles and Diseases; SNPs; and References.

Browsers are provided for exploring various vocabularies used in MGD (e.g. GO, MP and OMIM disease terms) and terms from these vocabularies are linked to relevant MGD annotated data. Genome browsing is accomplished with our installation of JBrowse (http://jbrowse.org), a JavaScript-based interactive genome browser with extensive features for navigation and track selection (13).

MGD offers additional batch methods for data querying and downloads for users wishing to retrieve data in bulk.

The Batch Query tool (http://www.informatics.jax.org/batch) (25–26) is used for retrieving bulk data about lists of genome features that can be typed in or uploaded as lists of gene symbols or gene IDs. Gene IDs from MGI, NCBI's GENE, Ensembl, VEGA, UniProt and other resources can be used. Users can select information they wish to retrieve, such as genome location, GO annotations, list of mutant alleles, MP annotations, RefSNP IDs and OMIM terms. Results are returned as a web display or in tab delimited text or Excel format.

MGD maintains an instance of BioMart containing two sets of data, mouse genes and genome features and mouse developmental gene expression (27). BioMart is a warehousing system that allows querying across BioMart instances. Thus, for example, MGD genes could be queried in conjunction with transcript IDs in the VEGA BioMart.

MGD access is also powered through MouseMine (http://www.mousemine.org)(28), an instance of InterMine that offers flexible querying, templates, iterative refinement of results and linking to other model organism InterMine instances. MouseMine contains many data sets from MGD, including genes and genome features, alleles, strains and annotations to GO, MP and OMIM.

MGD also provides a large set of regularly updated database reports via our FTP site (ftp://ftp.informatics.jax.org), and direct SQL access to a read-only copy of the database (contact MGI user support for an account). MGI User Support is also available to assist users in generating custom reports on request.

## CITING MGD

For a general citation of the MGI resource please cite this article. In addition, the following citation format is suggested when referring to data sets specific to the MGD component of MGI: Mouse Genome Database (MGD), Mouse Genome Informatics, The Jackson Laboratory, Bar Harbor, Maine (URL: http://www.informatics.jax.org) (Type in date (month, year) when you retrieved the data cited.).
